# Supplementary material for: AP‐3 vesicle uncoating occurs after HOPS‐dependent vacuole tethering
Source: EMBO J. 2020 Aug 25;39(20):e105117. doi: 10.15252/embj.2020105117 (PMC7560216; doi:10.15252/embj.2020105117)
Supplement: Supplementary file 1 — Appendix [file EMBJ-39-e105117-s001.pdf]

AP-3 vesicle uncoating occurs after HOPS-dependent vacuole tethering

Jannis Schoppe[1], Muriel Mari[2], Erdal Yavavli [1], Kathrin Auffarth[1], Margarita Cabrera[3], Stefan Walter[4], Florian Fröhlich[4,5], and Christian Ungermann[1,4,6]

Appendix

Table of Contents

Appendix Legend - Page 2

Appendix Figure S1 - Page 3

Appendix Figure S2 - Page 4

Appendix Table S1 - Page 5

Appendix Legends

Appendix Figure S1: Effect of Vps41 S-A mutation on AP-3 localization to mitochondria. Cells expressing either wild-type Vps41-GFP-Fis1 or the Vps41 S-A variant and Apl6- mCherry were grown at 30°C in YPG and kept in logarithmic phase. Just prior to imaging, the mitochondrial DNA was stained with DAPI as described in the methods. Localization of Apl6 and Vps41 was analysed by fluorescence microscopy. DIC image is shown in first column. Scale bar, 5 µm. The inset shows the indicated magnification. Scale bar in inset, 2 µm.

Appendix Figure S2: Quantification of AP-3 puncta at the mitochondrial surface. **(A)** Tethering of AP-3 vesicles to the mitochondrial surface in *age2Δ* background. Cells were grown at 30°C in YPG and kept in logarithmic phase. Localization of Apl5 and Vps41 was analysed in the indicated strains by fluorescence microscopy. DIC image is shown in first column. **(B)** Quantification of (A). Apl6-dots co-localizing with GFP signal were counted and plotted in percent of the total number of Apl6-dots ($n \geq 60$ cells). **(C)** AP-3 positive structures were counted and averaged per cell for all indicated strains. Box boundaries indicate 25% and 75% of the dataset, the middle line indicates the median and the small squares the mean. Whiskers correspond to 5% and 95% values while the small lines show the farthermost outliers. Only relevant changes of significance are indicated ($n \geq 60$ cells). Significance was determined with a two-tailed t-test (***, $p \leq 0.001$).

Appendix Table S1: Yeast strains used in this study.

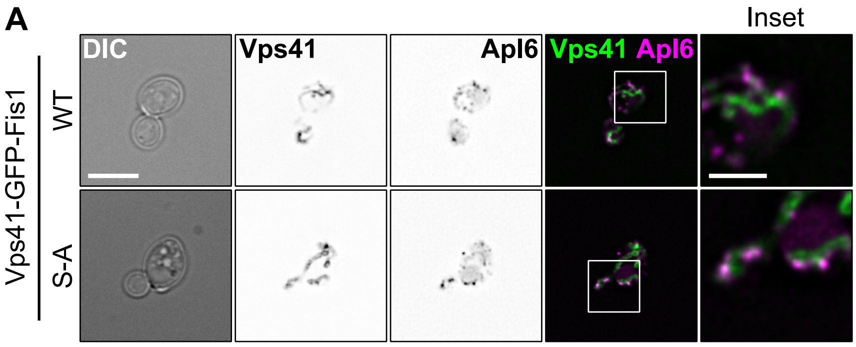
A
Inset
DIC
Vps41
Apl6
Vps41 Apl6
Vps41-GFP-Fis1
WT
S-A

Appendix Figure S2,
Schoppe et al.

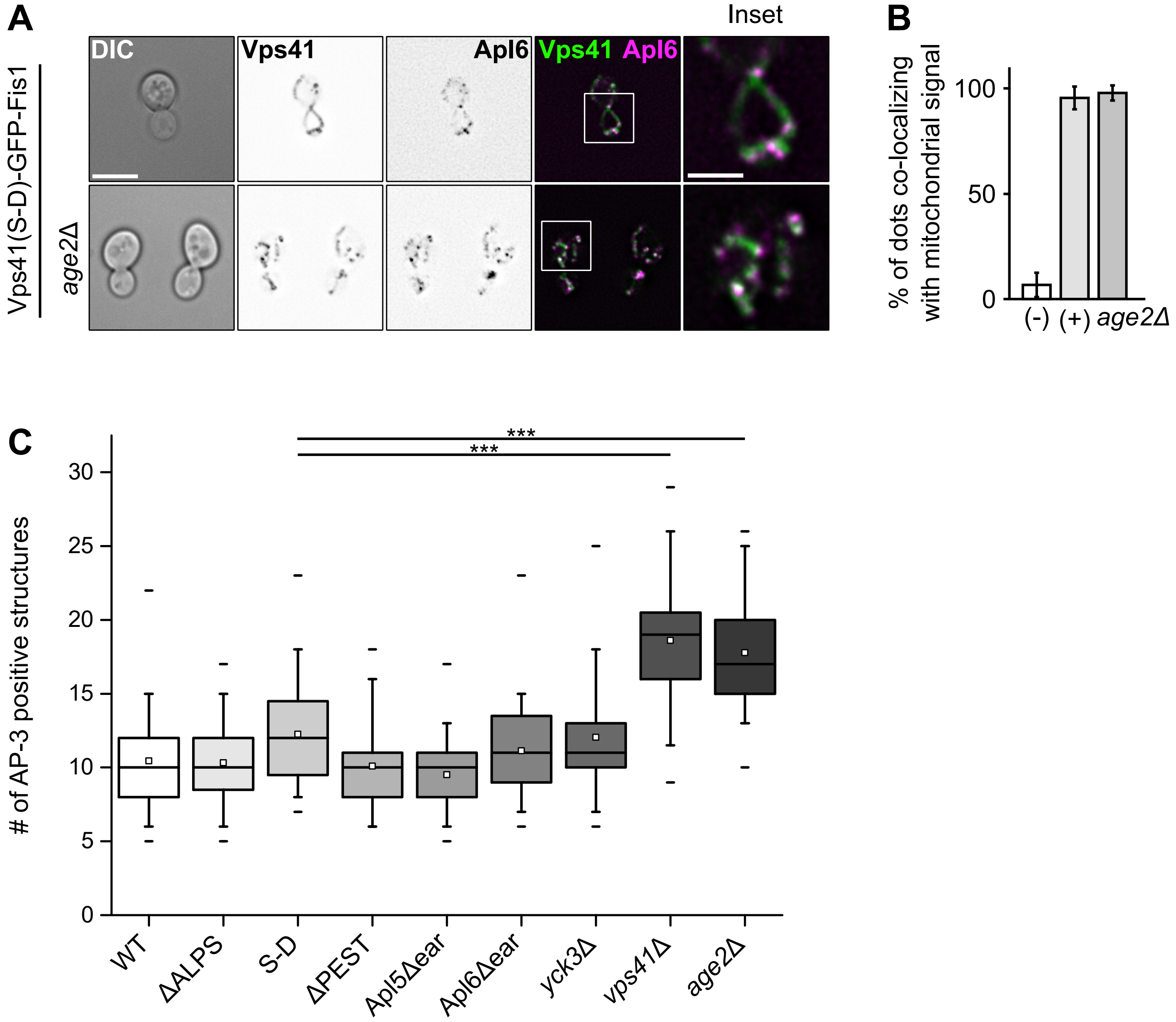

Appendix Table S1

	Genotype	Reference
CUY9908	MATa *his3*Δ1 *leu2*Δ0 *met15*Δ0 *ura3*Δ0 *vam3*Δ::kanMX *vam3*ts::MET15	This study
CUY11150	CUY9908 *VPS41*::mNeonGFP-hphNT1 *SEC7*::mCherry-natNT2	This study
CUY12575	CUY9908 *APL5*::mGFP-hphNT1 *SEC7*::mCherry-natNT2	This study
CUY12576	CUY9908 *VPS41*::mNeonGFP-hphNT1 *APL5*::mCherry-natNT2	This study
CUY6637	MATalpha *tor1-1 fpr1*::NAT *PMA1*-2xFKBP12::TRP1 pRS402::ADE *VPS41*-FRB-GFP::kanMX *VPS41*::URA-TEFpr *APL5*::mCherry-hphNT1	This study
CUY764	MATa *his3*Δ1 *leu2*Δ0 *met15*Δ0 *ura3*Δ0	Euroscarf
CUY9907	MATa *his3*Δ1 *leu2*Δ0 *met15*Δ0 *ura3*Δ0 *clc1*Δ::kanMX	Euroscarf
CUY469	MATa *his3*Δ1 *leu2*Δ0 *met15*Δ0 *ura3*Δ0 *yck3*Δ::kanMX	Euroscarf
CUY485	MATa *his3*Δ1 *leu2*Δ0 *met15*Δ0 *ura3*Δ0 *apl5*Δ::kanMX	Euroscarf
CUY12569	MATalpha *leu2*-3,112 *ura3*-52 *his3*-Δ200 *trp*-Δ901 *lys2*-801 *suc2*-Δ9 GAL *gcs1*Δ::natNT2	This study
CUY12317	MATalpha *leu2*-3,112 *ura3*-52 *his3*-Δ200 *trp*-Δ901 *lys2*-801 *suc2*-Δ9 GAL *age2*Δ::natNT2	This study
CUY12497	MATa *his3*Δ1 *leu2*Δ0 *met15*Δ0 *ura3*Δ0 *VPS41*::natNT2-GAL1pr	This study
CUY12499	MATa *his3*Δ1 *leu2*Δ0 *met15*Δ0 *ura3*Δ0 *URA3*::pRS406-VPS41pr-VPS41 *VPS41*::kanMX-GAL1pr *VPS41*::GFP-Fis1-hphNT1	This study
CUY12557	MATa *his3*Δ1 *leu2*Δ0 *met15*Δ0 *ura3*Δ0 *URA3*::pRS406-VPS41pr-VPS41 *VPS41*::kanMX-GAL1pr *VPS41*::GFP-Fis1-hphNT1 *APL6*::mCherry-natNT2	This study
CUY12503	MATa *his3*Δ1 *leu2*Δ0 *met15*Δ0 *ura3*Δ0 *URA3*::pRS406-VPS41pr-VPS41(S-A) *VPS41(S-A)*::GFP-Fis1-hphNT1	This study
CUY12559	MATa *his3*Δ1 *leu2*Δ0 *met15*Δ0 *ura3*Δ0 *URA3*::pRS406-VPS41pr-VPS41(S-A) *VPS41(S-A)*::GFP-Fis1-hphNT1 *APL6*::mCherry-kanMX	

Appendix Table S1

CUY12614	MATa *his3*Δ1 *leu2*Δ0 *met15*Δ0 *ura3*Δ0 *URA3*::pRS406-VPS41pr-*VPS41*ΔALPS *VPS41*ΔALPS::GFP-Fis1-hphNT1 *APL6*::mCherry-kanMX	This study
CUY12501	MATa *his3*Δ1 *leu2*Δ0 *met15*Δ0 *ura3*Δ0 *URA3*::pRS406-VPS41pr-VPS41ΔPEST *VPS41*ΔPEST::GFP-Fis1-hphNT1	This study
CUY12558	MATa *his3*Δ1 *leu2*Δ0 *met15*Δ0 *ura3*Δ0 *URA3*::pRS406-VPS41pr-VPS41ΔPEST *VPS41*ΔPEST::GFP-Fis1-hphNT1 *APL6*::mCherry-kanMX	This study
CUY12505	MATa *his3*Δ1 *leu2*Δ0 *met15*Δ0 *ura3*Δ0 *URA3*::pRS406-VPS41pr-VPS41(S-D) *VPS41*(S-D)::GFP-Fis1-hphNT1	This study
CUY12560	MATa *his3*Δ1 *leu2*Δ0 *met15*Δ0 *ura3*Δ0 *URA3*::pRS406-VPS41pr-VPS41(S-D) *VPS41*(S-D)::GFP-Fis1-hphNT1 *APL6*::mCherry-kanMX	This study
CUY12564	MATa *his3*Δ1 *leu2*Δ0 *met15*Δ0 *ura3*Δ0 *URA3*::pRS406-VPS41pr-VPS41(S-D) *VPS41*(S-D)::GFP-Fis1-hphNT1 *APL5*::ΔEAR-mCherry-natNT1	This study
CUY12565	MATa *his3*Δ1 *leu2*Δ0 *met15*Δ0 *ura3*Δ0 *URA3*::pRS406-VPS41pr-VPS41(S-D) *VPS41*(S-D)::GFP-Fis1-hphNT1 *APL6*::ΔEAR-mCherry-natNT1	This study
CUY12566	MATa *his3*Δ1 *leu2*Δ0 *met15*Δ0 *ura3*Δ0 *URA3*::pRS406-VPS41pr-VPS41(S-D) *VPS41*(S-D)::GFP-Fis1-hphNT1 *age2*Δ::kanMX *APL6*::mCherry-natNT2	This study
CUY12567	MATa *his3*Δ1 *leu2*Δ0 *met15*Δ0 *ura3*Δ0 *URA3*::pRS406-VPS41pr-VPS41(S-D) *VPS41*(S-D)::GFP-Fis1-hphNT1 *yck3*Δ::kanMX *APL6*::mCherry-natNT2	This study
CUY12568	MATa *his3*Δ1 *leu2*Δ0 *met15*Δ0 *ura3*Δ0 *URA3*::pRS406-VPS41pr-VPS41(S-D) *VPS41*(S-D)::GFP-Fis1-hphNT1 *vps41*Δ::kanMX *APL6*::mCherry-natNT2	This study
CUY12609	MATalpha *leu2*-3,112 *ura3*-52 *his3*-Δ200 *trp*-Δ901 *lys2*-801 *suc2*-Δ9 GAL *APL5*::mGFP-hphNT1	This study
CUY12572	MATalpha *leu2*-3,112 *ura3*-52 *his3*-Δ200 *trp*-Δ901 *lys2*-801 *suc2*-Δ9 GAL *age2*Δ::natNT2 *APL5*::mGFP-hphNT1	This study
CUY12573	MATalpha *leu2*-3,112 *ura3*-52 *his3*-Δ200 *trp*-Δ901 *lys2*-801 *suc2*-Δ9 GAL *gcs1*Δ::natNT2 *APL5*::mGFP-hphNT1	This study
CUY12610	MATalpha *leu2*-3,112 *ura3*-52 *his3*-Δ200 *trp*-Δ901 *lys2*-801 *suc2*-Δ9 GAL *APL5*::mGFP-hphNT1 *SEC7*::mCherry-natNT2	This study

Appendix Table S1

CUY12611	MATalpha *leu2*-3,112 *ura3*-52 *his3*-Δ200 *trp*-Δ901 *lys2*-801 *suc2*-Δ9 GAL *age2*Δ::natNT2 *APL5*::mGFP-hphNT1 *SEC7*::mCherry-natNT2	This study
CUY12318	MATa *his3*Δ1 *leu2*Δ0 *met15*Δ0 *ura3*Δ0 *APL5*::TurboID-V5-kanMX4	This study
CUY12312	MATa *his3*Δ1 *leu2*Δ0 *met15*Δ0 *ura3*Δ0 *URA3*::pRS406-VPS41pr-VPS41(S-D) *VPS41*(S-D)::GFP-Fis1-hphNT1 *APL5*::TurboID-V5-kanMX4	This study
CUY12667	MATalpha *leu2*-3,112 *ura3*-52 *his3*-Δ200 *trp*-Δ901 *lys2*-801 *suc2*-Δ9 GAL *age2*Δ::natNT2 *URA3*::pRS406-AGE2pr-AGE2-AGE2term	This study
CUY12668	MATalpha *leu2*-3,112 *ura3*-52 *his3*-Δ200 *trp*-Δ901 *lys2*-801 *suc2*-Δ9 GAL *age2*Δ::natNT2 *URA3*::pRS406-AGE2pr-AGE2-AGE2term *APL5*::mGFP-hphNT1	This study
CUY12669	MATalpha *leu2*-3,112 *ura3*-52 *his3*-Δ200 *trp*-Δ901 *lys2*-801 *suc2*-Δ9 GAL *age2*Δ::natNT2 *URA3*::pRS406-AGE2pr-AGE2(R52K)-AGE2term	This study
CUY12670	MATalpha *leu2*-3,112 *ura3*-52 *his3*-Δ200 *trp*-Δ901 *lys2*-801 *suc2*-Δ9 GAL *age2*Δ::natNT2 *URA3*::pRS406-AGE2pr-AGE2(R52K)-AGE2term *APL5*::mGFP-hphNT1	This study
CUY12671	MATalpha *leu2*-3,112 *ura3*-52 *his3*-Δ200 *trp*-Δ901 *lys2*-801 *suc2*-Δ9 GAL *gcs1*Δ::natNT2 *URA3*::pRS406-GCS1pr-GCS1-GCS1term	This study
CUY12672	MATalpha *leu2*-3,112 *ura3*-52 *his3*-Δ200 *trp*-Δ901 *lys2*-801 *suc2*-Δ9 GAL *gcs1*Δ::natNT2 *URA3*::pRS406-GCS1pr-GCS1-GCS1term *APL5*::mGFP-hphNT1	This study
CUY12673	MATalpha *leu2*-3,112 *ura3*-52 *his3*-Δ200 *trp*-Δ901 *lys2*-801 *suc2*-Δ9 GAL *gcs1*Δ::natNT2 *URA3*::pRS406-GCS1pr-GCS1(R54K)-GCS1term	This study
CUY12674	MATalpha *leu2*-3,112 *ura3*-52 *his3*-Δ200 *trp*-Δ901 *lys2*-801 *suc2*-Δ9 GAL *gcs1*Δ::natNT2 *URA3*::pRS406-GCS1pr-GCS1(R54K)-GCS1term *APL5*::mGFP-hphNT1	This study
CUY12689	MATalpha *leu2*-3,112 *ura3*-52 *his3*-Δ200 *trp*-Δ901 *lys2*-801 *suc2*-Δ9 GAL *GCS1*::kanMX-TEFpr	This study
CUY12690	MATalpha *leu2*-3,112 *ura3*-52 *his3*-Δ200 *trp*-Δ901 *lys2*-801 *suc2*-Δ9 GAL *GCS1*::kanMX-TEFpr *APL5*::mGFP-hphNT1	This study
CUY12691	MATalpha *leu2*-3,112 *ura3*-52 *his3*-Δ200 *trp*-Δ901 *lys2*-801 *suc2*-Δ9 GAL *AGE2*::kanMX-TEFpr	This study

Appendix Table S1

CUY12692	MATalpha *leu2*-3,112 *ura3*-52 *his3*-Δ200 *trp*-Δ901 *lys2*-801 *suc2*-Δ9 GAL *AGE2*::kanMX-TEFpr *APL5*::mGFP-hphNT1	This study
